# Characterization of saline dust emission resulted from Urmia Lake drying

**DOI:** 10.1186/s40201-015-0238-3

**Published:** 2015-11-28

**Authors:** Akbar Gholampour, Ramin Nabizadeh, Mohammad Sadegh Hassanvand, Hasan Taghipour, Shahrokh Nazmara, Amir Hossein Mahvi

**Affiliations:** Center for Air Pollution Research, Institute for Environmental Research, Tehran University of Medical Sciences, Tehran, Iran; School of Public Health, Tabriz University of Medical Sciences, Tabriz, Iran; School of Public Health, Tehran University of Medical Sciences, Tehran, Iran; Center for Solid Waste Research, Institute for Environmental Research, Tehran University of Medical Sciences, Tehran, Iran; National Institute of Health Research, Tehran University of Medical Sciences, Tehran, Iran

**Keywords:** Urmia Lake, Saline dust, Particulate matters, Water-soluble ions, Source identification, Ions correlation

## Abstract

Compared with common dust storms, saline dust storms transport high concentrations of fine-grain saline and alkaline material. The saline dust storm differs from common dust storm, especially considering the sources of the suspended particulate matter (PM), chemical composition, grain size, and circulation processes. Atmospheric particulate matters (TSP, PM_10_, PM_2.5_, and PM_1_) and their water-soluble ions were concurrently measured at two sites located at north and southeast part of Urmia lake from January 2013 to September 2013. Particulate matters (PMs) were measured using high volume sampler and HAZ-DUST EPAM-5000 particulate air monitors. In both of the sampling sites, the highest concentration of PM was observed during the summer season (521.6, 329.1, 42.6, and 36.5 for TSP, PM_10_, PM_2.5_, and PM_1_, respectively). A total of 11 inorganic water-soluble ions in the TSP and PM_10_ were identified by ion chromatography (IC). No statistically significant difference was found between PM’s ions concentrations of two sampling sites. The average of the total measured water-soluble ions in the sampling sites was 28.75 ± 12.9 μg/m^3^ (11.9 ± 4.8% of total TSP mass) for TSP and 14.65 ± 7.1μg/m^3^ (8.7 ± 4.4 of total PM_10_ mass) for PM_10_. Among all detected ions, sulfate was the dominant constituent followed by nitrate and sodium. This study showed that the water soluble salts compose 3–20% of the total mass of TSP and PM_10_. The PCA analysis showed that saline particulates formed from Urmia lake bed were the dominant source (57.6 %) of TSP. In addition, saline particulates together with crustal materials resulted from resuspension were the main source (59.9%) of PM_10_.

## Background

Dust Storm phenomenon could change global climate and affect the economy and quality of human life [1]. In addition to contribution of the formation of gypcrete and calcrete, dust may lead to accumulation of more water soluble salts in soil profiles and thus contribute to salinization [2]. The saline dust storm differs from common dust storm, especially considering the sources of the suspended particulate matter (PM), chemical composition, grain size, and circulation processes. Saline dust storms are defined as “a kind of environmental disaster phenomenon in arid and semiarid regions that has been caused by dust deflated from the salt-rich sediments of dried lake beds and strongly salinized soils on the margins of lake floors” [3, 4]. Such a phenomena has been registered in many parts of the world, including the Aral Sea region in Kazakhstan and Uzbekistan, Lake Balkhash region in Kazakhstan, the Inner Mongolian region of China, southeastern Australia, and many other regions with arid and semiarid climate [5]. It was cleared that due to salt accumulation, sparse or absent of vegetation, and easily occurrences of wind erosion, these surface sediments will form a sleazy texture [3].

Compared with common dust storms, saline dust storms transport high concentrations of fine-grain saline and alkaline material, such as sodium sulfate, sodium chloride, and other potentially toxic components which could be threaten to the ecological security and humans health in the arid regions [3, 6, 7].

As mentioned above, chemical composition of saline dust storms is concerned to the characterization of region soil properties, and this redistribution leads to dual effects depending on the nature of transported material. Based on studies, saline dust storms are rich in CaCl_2_, NaCl, MgCl_2_ and other chloride particles, which can be suspended in the atmosphere for long periods of time [8].

**Fig. 1:**
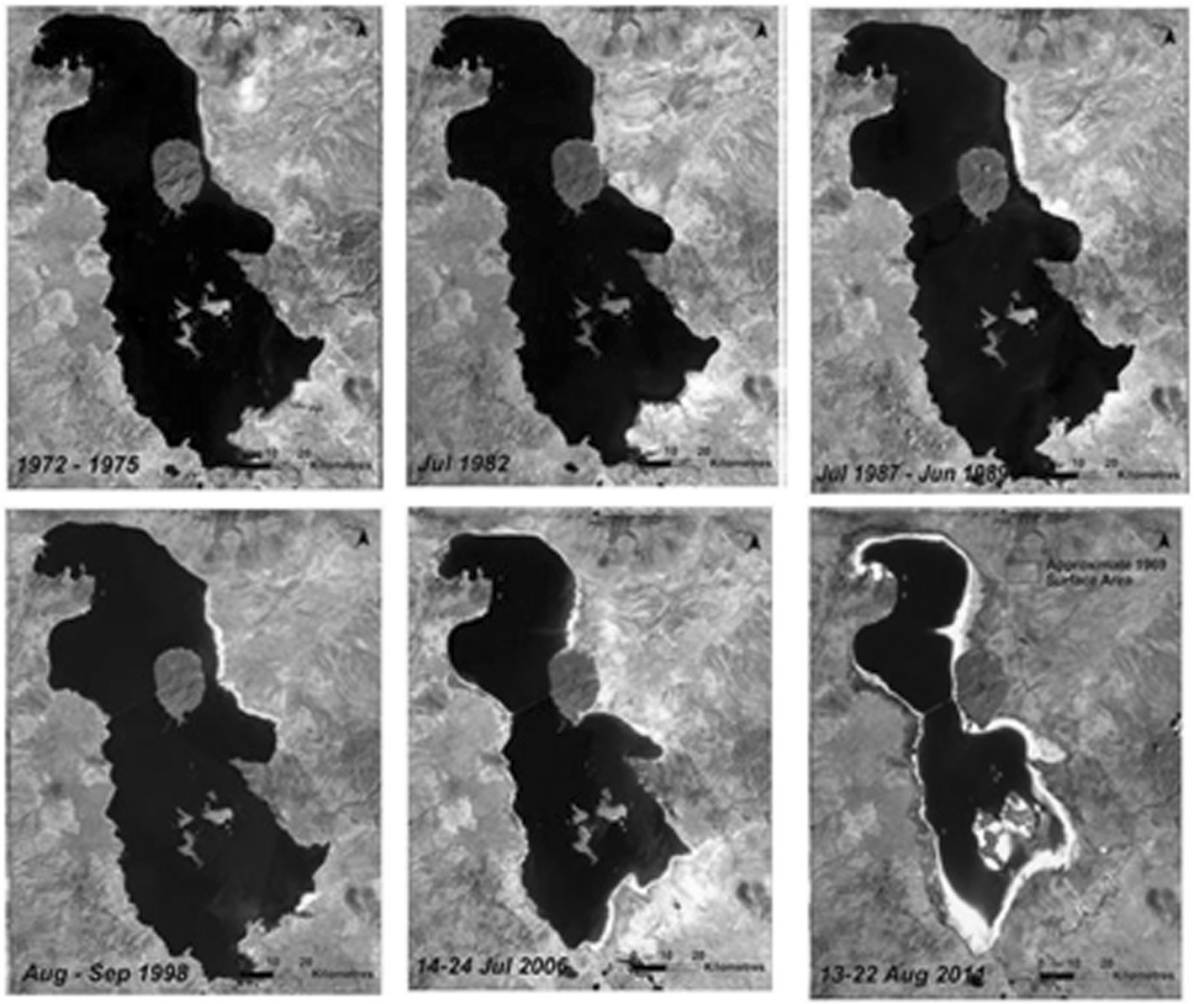
Decreasing area of Urmia Lake since 1972 to 2011 shown in LANDSAT images (Pengra, 2012)

Urmia lake, located in northwest of Iran, is the second great saline lake in the world and the largest lake in Iran. In recent decade, the water level at Urmia lake has fallen more than 5 meters and the concentration of salt in this lake has increased from 185 to 220 g/L [9]. In addition to decrease in water level and increase in salt concentration, the unique ecosystem of the lake is being destroyed. As a result, a salt desert would be created with an area of more than 5000 km^2^ (Fig. 1), overlaid with a 50–60 cm thick salt deposits [10]. In the warm season, wind could carry out these salts and transport those to adjacent areas as far as 300 km. The transported PM could damage agricultural lands, pollute the ecosystem and cause variety of diseases in nearby urban and rural areas [9].

Hitherto, no study has been conducted about atmospheric PM in Urmia Lake bed. The present study was therefore carried out to determine the mass levels of total suspended particulate (TSP), PM_10_, PM_2.5_ and PM_1_ (aerodynamic diameter smaller than10, 2.5 and 1μm, respectively) along with the variations of water-soluble ionic species associated with TSP and PM_10_ in the floor of Urmia Lake.

## Methods

### Study area, sampling sites and schedule

**Fig. 2:**
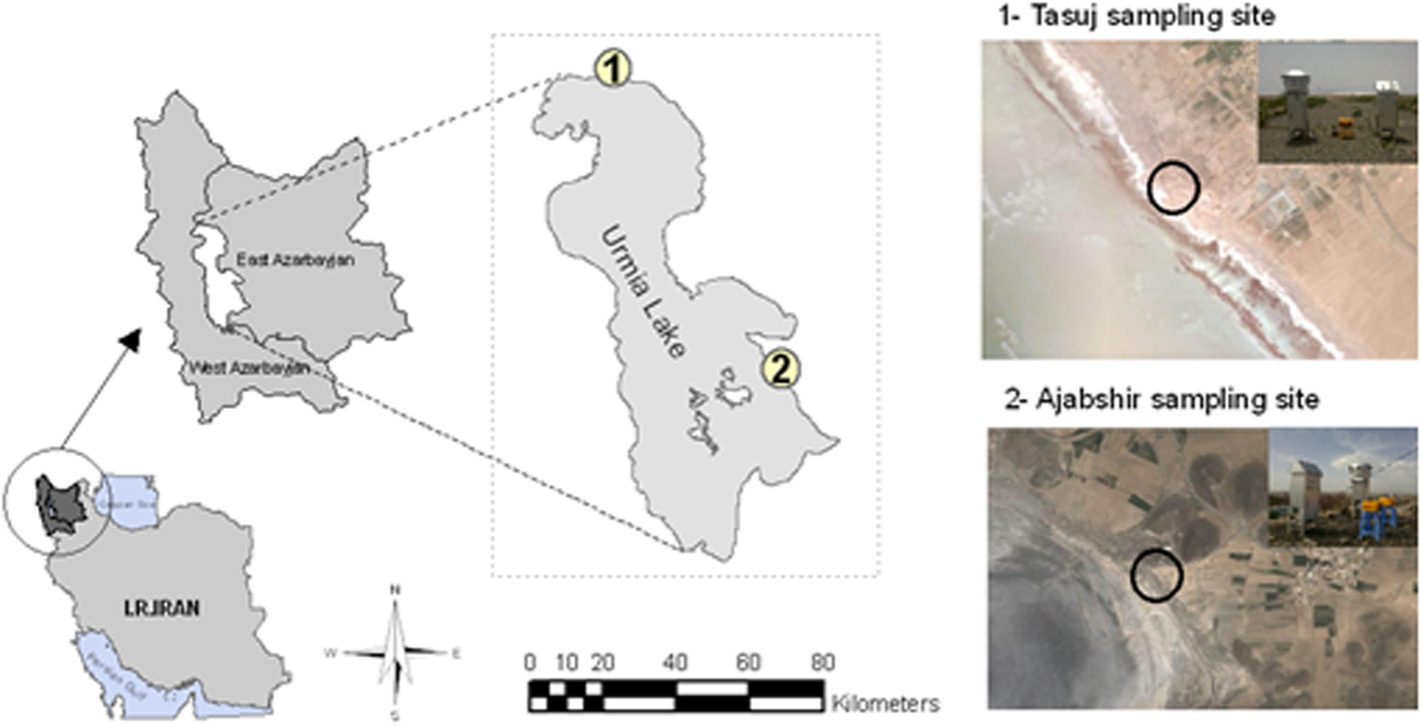
Location of study area and sampling sites

Based on the region wind direction, two sites were selected (Fig. 2); 1) Tasuj site, located in the north of Urmia lake (38° 13' 17.8" N - 45° 24' 38.5" E), and 2) Ajabshir site, located in the southeast of Urmia lake (37° 31' 12.9" N - 45° 47' 53.0" E). The level of TSP, PM_10_, PM_2.5_ and PM_1.0_ were measured 8 times every season in the period of January 2013 to September 2013. In total, 48 samples of TSP and PM_10_ were collected and analyzed during the study period.

### PMs measurement

TSP and PM_10_ samples were collected by two high volume samplers manufactured by Graseby–Andersen at flow rates of 1.13–1.41 m^3^/min for 24 h. Both TSP and PM_10_ were collected on a 20.3cm _X_ 25.4cm Whatman glass micro fiber filter. Before and after sampling, filters were set under 40% relative humidity (RH) at 25°C for over 48h, afterward at room condition for 2 h; then were weighed three times using an A&D electronic balance with the reading precision of 0.1mg. PM_2.5_ and PM_1.0_ were measured using two portable HAZ-DUST EPAM-5000 particulate air monitors.

### Chemical analysis

For water soluble ions analysis, one quarter of each filter was placed in a glass vial and then 40 mL ultra pure water (specific resistance ≥18 Ω cm) was added. The vials were shaken for 2h, and subsequently were ultra-sonicated for 30min. The extracted solutions were filtered through a micro porous membrane (with the pore size 0.45 μm) [11]. An ion chromatograph (Metrohm 850 Professional IC, Switzerland) with operating flow rate of 0.7 mL/min was used to analyze water-soluble ions. Field and laboratory blanks and spiked samples were analyzed along with the PM samples. For all ions, method detection limits (MDLs) were calculated by adding three standard deviations of the blank readings to the average of five replicates of the blank. The obtained MDLs and the recovery efficiencies for water-soluble ions are presented in Table 1.

**Table 1:** MDLs and recovery efficiencies for water- soluble ions

Water-soluble ion	*Na* ^+^	*NH*_4_^+^	*K* ^+^	*Mg* ^2+^	*Ca* ^2+^	*F* ^−^	*Cl* ^−^	*NO*_2_^−^	*NO*_3_^−^	*SO*_4_^2 −^	*PO*_4_^2 −^
MDLs^a^ (ng/mL)	95	6.3	4.4	12.5	5.3	3.7	6.5	3.6	52	45	6.1
MDLs^a^ (μg/m^3^)	0.0025	0.0002	0.0001	0.0003	0.0001	0.0001	0.0002	0.0001	0.0014	0.0012	0.0002
Recovery efficiencies (%)	97–103	78–106	101–103	112–133	109–122	105–107	99–103	100–117	98–104	102–104	95–106

^a^Based on 3σ blank filters (*n* = 5)

### Size and morphology

To determine the morphology and the elemental composition of the collected particles, TSP and PM_10_ samples were analyzed separately using the SEM-EDX system at the Razi Metallurgical Research Center.

### Data analysis

Data were analyzed (with SPSS20 statistical software, SPSS Inc.) by means of the linear regression (for correlation coefficients among water-soluble ions), the bivariate correlations (to quantify the relation between the elemental concentrations), dimension reduction factor (for the quality principal components (PCs) of TSP and PM_10_), and multivariate test (to quantify significance different between ions concentrations in Tasuj site vs. Ajabshir site). Differences and correlations were considered significant at the 0.05 level.

Meteorological data were obtained from the national climatic data center [12] and East Azerbaijan Meteorological Organization. The obtained data were examined for the missing values and outliers, and then were entered into WRPLOT View Freeware 7.0.0 to plot the wind-rose. The concentrations of PM were analyzed using Microsoft Excel 2010.

## Results and discussion 

### Meteorological dates

**Fig. 3:**
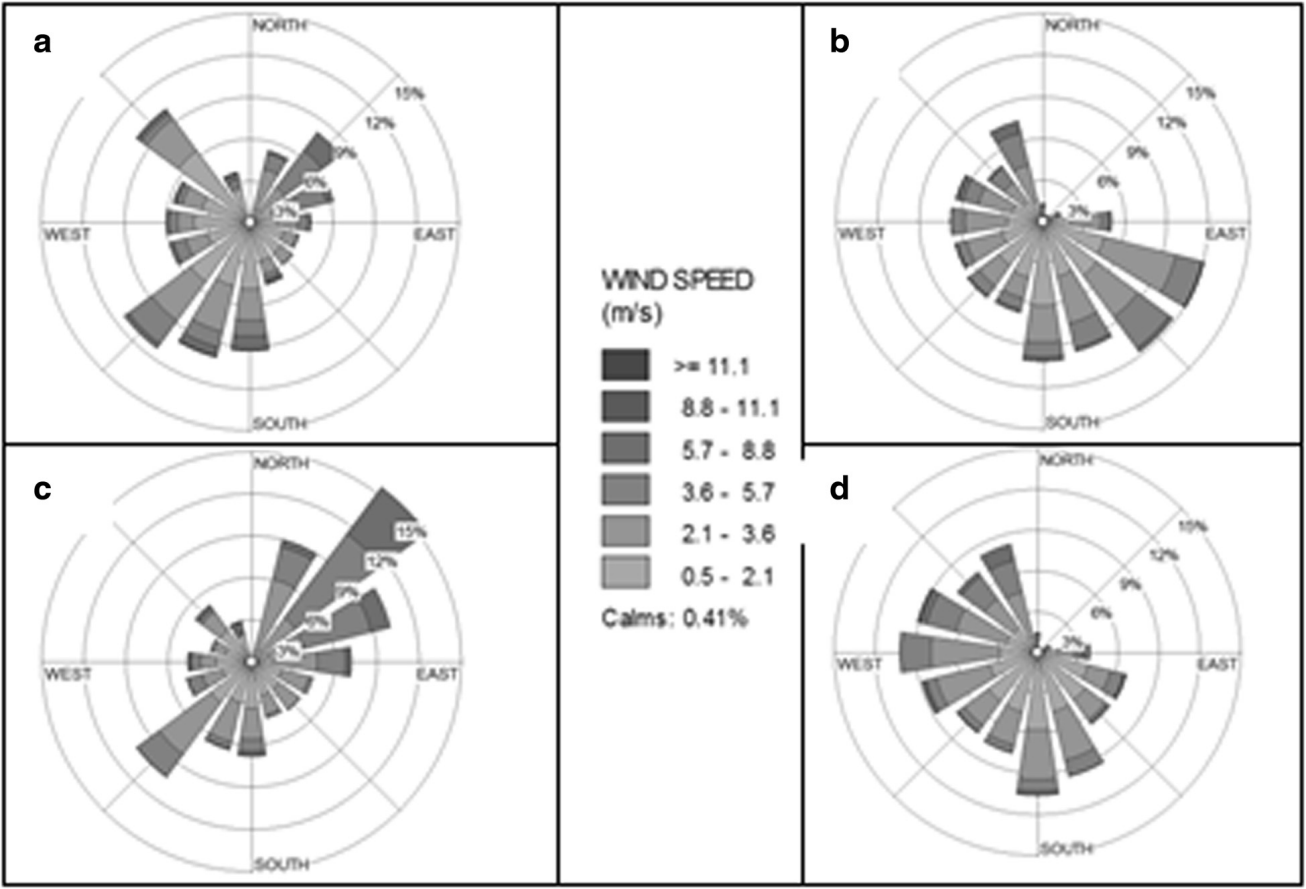
Annual wind rose plots during the year of 2012 for (**a**) Tasuj and (**b**) Ajabshir and wind rose plots during June to January for (**c**) Tasuj and (**d**) Aajabshir

Based on the collected meteorological dates, in both sampling sites, February was the coldest month with the monthly mean temperature of −1.0°C, while August was the warmest month with the mean temperature of 28.0°C. Also the RH varied from 25 to 72%. Dates of wind speed and the direction in both sampling sites revealed that in Ajabshir township, seasonal mean wind speed were 2.8, 2.72, 2.48 and 2.12 m/s, while in the Tasuj township were 2.36, 3.28, 3.25 and 1.97 m/s in winter, spring, summer and autumn, respectively. Annual wind rose plots for both sampling sites are shown in Fig. 3.

In Tasuj Township, the annual prevailing wind blew from South and South West with the speed of 0.5–13.6 m/s. The annual mean wind speed was 2.57 m/s. Calm wind (0 m/s) frequencies were 0.13, 0.6, 1.0, and 2.24% in spring, summer, autumn and winter, respectively. In Ajabshir Township, wind speed varied from 0.5 to 11.12 m/s and annual mean wind speed was 2.57 m/s. In this region the annual prevailing wind blew from South and South East. Also, calm wind (0 m/s) frequencies were 0.5, 0.5, 2.9, and 1.0% in spring, summer, autumn and winter, respectively.

It was cleared that a drought associated with strong wind is regarded as the climatic background for the formation of dust storms [13, 14]. With respect to meteorological dates, June to September have the greatest potential to create dust storm, therefore, in order to predict the direction of probable saline dust storm, wind rose was plotted for mentioned months

(Fig. 3). As it can be seen in Fig. 3, in Tasuj sampling site prevailing wind blew from North East and South West during dry season; and in Ajabshir Township prevailing wind was from South to North and from

### The PM mass concentrations and relations

Descriptive statistics of PM mass concentrations and the ratio of PMs’ species in two sampling sites are presented in Table 2. According to Table 2, the average concentrations of TSP, PM_10_, PM_2.5_, and PM_1_ in Tasuj sampling site were 230.1 ± 71.7, 140.3 ± 37.2, 28.3 ± 6.3, and 23.0 ± 5.6 μg/m^3^, respectively. In Ajabshir sampling site the average concentrations were 291.4 ± 140.3, 220.1 ± 108.6, 31.8 ± 8.7, and 27.4 ± 7.6 μg/m^3^ for TSP, PM_10_, PM_2.5_, and PM_1_, respectively. In two sampling sites, the highest concentration of PM was observed during the summer season followed by spring.

**Table 2:** Descriptive statistics for 24- hour PM mass concentrations (μg/m^3^) and the ratio of PMs’ species in Ajabshir and Tasuj sampling sites

Sampling sites		PM concentrations (μg/m^3^)				The ratio of PMs’ species			
		TSP	PM_10_	PM_2.5_	PM_1_	PM_10_/TSP	PM_2.5_/PM_10_	PM_1_/PM_10_	PM’_1_/PM_2.5_
Tasuj	Min	150.80	102.10	22.25	16.05	0.38	0.16	0.13	0.72
	Max	338.80	194.80	36.50	29.46	0.90	0.26	0.21	0.89
	Average	230.15	140.34	28.35	22.99	0.64	0.21	0.17	0.81
	SD	71.74	37.17	6.27	5.64	0.19	0.04	0.03	0.06
	Median	211.10	130.22	26.72	22.85	0.63	0.20	0.16	0.81
Ajabshir	Min	168.19	95.94	22.50	18.60	0.57	0.10	0.10	0.80
	Max	521.60	329.15	42.60	36.50	0.96	0.24	0.19	0.96
	Average	291.36	220.08	31.84	27.45	0.75	0.17	0.14	0.86
	SD	140.33	108.64	8.69	7.57	0.16	0.05	0.04	0.06
	Median	261.57	230.00	30.55	28.05	0.74	0.16	0.13	0.86

Also, Table 2 represents the ratio of PMs’ species in two sampling sites. The average of PM_10_/TSP ratio in Ajabshir site (0.75) was higher than those for Tasuj site (0.64). Also the PM_2.5_/PM_10_ ratio for Ajabshir and Tasuj sampling sites ranged between 0.10–0.24 and 0.16–0.16, respectively. There was no significance difference between PM’s ratios obtained from Tasuj and Ajabshir sampling sites (Wilks’ Lambda value = 0.73, F = 0.65 and Pvalue = 0.65). Compared to our previous study on Tabriz urban and industrial suburban PM ratios, PM_2.5_/PM_10_ in Urmia lake region was smaller than urban (0.48) and industrial zone (0.38) of Tabriz [15]. It seems due to significant differences in particle sources and also some geographical and meteorological conditions the obtained results from one study could not be directly compared with the findings of other studies.

### Ionic composition of PM

The statistical analysis of water soluble ions concentration in the TSP and PM_10_ showed that there was no significant difference between PM’s ions concentrations at Tasuj and Ajabshir sites (Wilks’ Lambda value = 0.022, F = 4.35 and Pvalue = 0.36 for TSP and Wilks’ Lambda value = 0.019 F = 5.1 and Pvalue = 0.33 for PM_10_). Therefore, the concentrations of water soluble ions were reported together as Urmia lake PM’s ionic characterization.

**Fig. 4:**
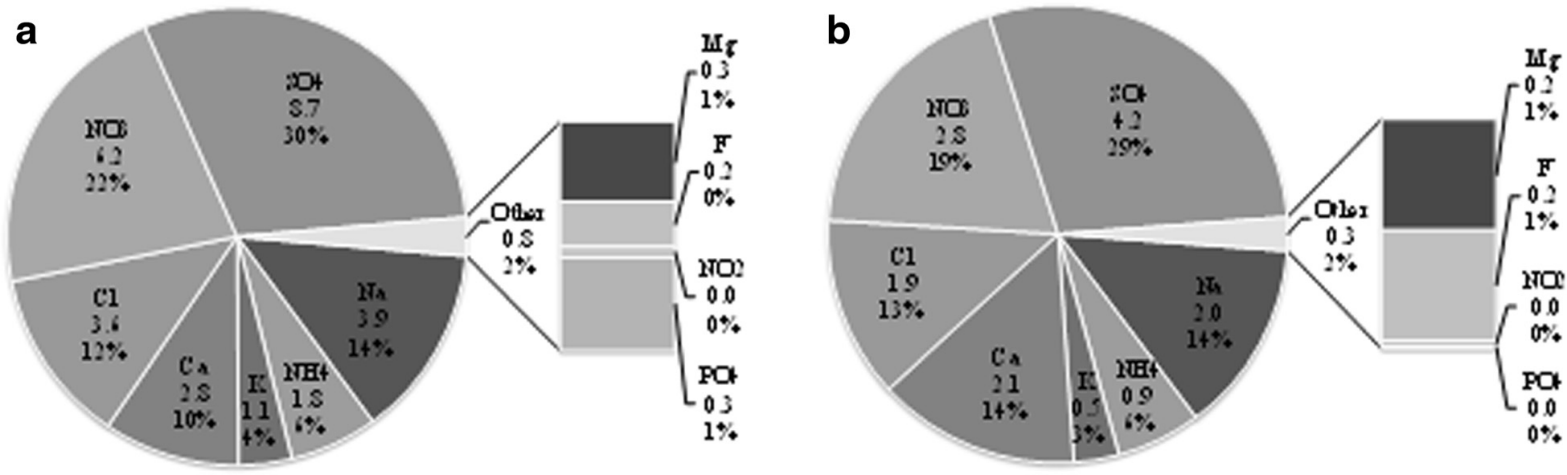
Water-soluble ions concentration in TSP (**a**) and PM_10_ (**b**) collected from Urmia Lake

The descriptive statistics of water-soluble ions concentration in TSP and PM_10_ collected from Urmia lake region are given in Table 3; expressed by cubic meter of sampled air (species in mass per volume units: μg/m^3^) and by mg per g of sampled particles (species in mass/mass units: mg/g of TSP and PM_10_). Also, the average of water-soluble ions concentration in TSP and PM_10_ are presented in Fig. 4(a and b); expressed by cubic meter of sampled air (μg/m^3^) and percentage of species in the measured water soluble ions mass concentration.

**Table 3:** Elemental analysis of Urmia lake TSP (PM_10_) (μg/m^3^and mg/g) (*n* = 48)

	μg/m^3^					mg/g				
	Min	Max	Median	Mean	SD	Min	Max	Median	Mean	SD
Na^+^	1.527 (1.114)	9.657 (4.524)	3.596 (1.793)	3.908 (1.994)	2.225 (0.875)	3.121 (2.720)	33.267 (25.982)	15.364 (12.817)	16.417 (12.858)	8.306 (6.966)
NH_4_^+^	0.184 (0.059)	4.664 (1.526)	1.645 (0.973)	1.787 (0.872)	1.288 (0.496)	0.516 (0.419)	15.701 (12.366)	5.628 (4.591)	7.357 (5.333)	4.701 (3.856)
K^+^	0.229 (0.091)	1.700 (0.970)	1.195 (0.433)	1.074 (0.466)	0.476 (0.251)	0.438 (0.292)	9.142 (8.016)	4.457 (2.503)	4.753 (3.120)	2.563 (2.232)
Mg^2+^	0.164 (0.094)	0.567 (0.267)	0.237 (0.157)	0.270 (0.166)	0.117 (0.052)	0.379 (0.339)	1.572 (1.645)	1.189 (1.077)	1.130 (1.021)	0.403 (0.432)
Ca^2+^	1.822 (1.204)	4.769 (3.540)	2.687 (2.044)	2.763 (2.090)	0.842 (0.688)	5.533 (3.300)	22.560 (20.125)	12.349 (12.828)	12.006 (12.738)	5.068 (5.048)
F^−^	0.000 (0.000)	0.489 (0.680)	0.014 (0.010)	0.152 (0.166)	0.211 (0.251)	0.000 (0.001)	2.481 (4.808)	0.072 (0.073)	0.555 (0.874)	0.805 (1.608)
Cl^−^	2.227 (1.220)	5.506 (4.267)	3.307 (1.425)	3.553 (1.876)	1.223 (0.929)	4.350 (2.189)	29.619 (24.506)	14.420 (10.924)	15.316 (11.493)	6.726 (5.895)
NO_2_^−^	0.000 (0.000)	0.114 (0.039)	0.024 (0.005)	0.038 (0.009)	0.040 (0.012)	0.000 (0.001)	0.639 (0.194)	0.151 (0.048)	0.163 (0.052)	0.183 (0.057)
NO_3_^−^	1.334 (0.860)	13.281 (7.948)	5.174 (2.391)	6.199 (2.814)	4.251 (2.131)	5.800 (5.012)	62.317 (45.650)	27.651 (10.547)	25.539 (16.632)	16.400 (13.801)
SO_4_^2−^	2.400 (1.563)	20.572 (11.406)	6.014 (4.311)	8.694 (4.201)	6.346 (2.795)	9.119 (10.308)	79.699 (65.508)	36.516 (18.353)	34.815 (23.182)	21.675 (14.892)
PO_4_^2−^	0.000 (0.000)	0.830 (0.031)	0.184 (0.000)	0.316 (0.003)	0.332 (0.009)	0.001 (0.001)	3.839 (0.216)	0.923 (0.002)	1.250 (0.020)	1.361 (0.062)
Sum	15.595 (8.479)	49.267 (34.029)	24.080 (12.839)	28.753 (14.657)	12.941 (7.109)	30.176 (27.513)	227.127 (195.443)	124.526 (74.120)	119.301 (87.322)	48.723 (44.092)

The average of the total measured water-soluble ions in TSP was 28.75 ± 12.9 μg/m^3^ (11.9 ± 4.8% of total TSP mass) and in PM_10_ was 14.65 ± 7.1μg/m^3^ (8.7 ± 4.4 of total PM_10_ mass). As it can be seen in Fig. 4, about 90% of total detected water-soluble ions mass in PM_10_ included by *S0*_4_^2 −^ (29%), *NO*_3_^−^ (21%), *Na* + (14%), *Cl*^−^(13%), and *Ca*^2+^(100%) in TSP, and ***SO***_4_^2 −^ (29%), *NO*_3_^−^ (20%), *Na*^+^(15%), *Cl*^−^(12%), and *Ca*^2+^(12%). Among all detected ions, sulfate was the dominant constituent followed by nitrate and sodium. These results are in agreement with the study by Hassanzadeh et al. [16] which Na^+^, K^+^, Ca^2+^, Li^+^, and Mg^2+^ were the main cations, while Cl^−^, SO_4_^2 −^, and HCO_3_^−^ were the main anions in Urmia lake’s water. The Urmia lake is a hyper-saline lake and the concentrations of Na^+^ and Cl^−^ were roughly 4 times the concentration of natural seawater [16]. High concentrations of Na^+^ and Cl^−^ (especially for TSP) could be caused by the higher and persistent on-shore winds which create abundant sea water droplets and marine aerosols.

Our study show that the water soluble salts compose 3–20% of the total mass of TSP and PM_10_, while Abuduwaili et al. [3] stated that in the Ebinur region, the soluble salts compose 10–25% of the total mass of the saline dust, and salts are predominantly represented by sodium and calcium chlorides and sulfates. Gholampour et al. [15] reported that in the Tabriz urban and suburban region, that are the near to the Urmia lake, water soluble ions accounted for approximately 20 ± 10% of total TSP mass and 25 ± 12 of total PM_10_ mass. Low percentage of total ions in the PM mass could be due to the small amount of secondary ion, especially non sea salt sulfate and ammonium, in the PM of Urmia lake bed.

Despite of higher PM and ions concentration during warm season, the comparison of mass percentage of water soluble ions in the ambient air PM of Urmia lake during cold and warm seasons showed that there was no significant difference between the mass percentage of PM’s ions in the various seasons (Wilks’ Lambda value = 0.4, F = 1.64 and Pvalue = 0.2). Therefore, it could be concluded that in the Urmia lake region, the sources of PMs are same during various seasons.

### Size and morphology of aerosol particles

**Fig. 5:**
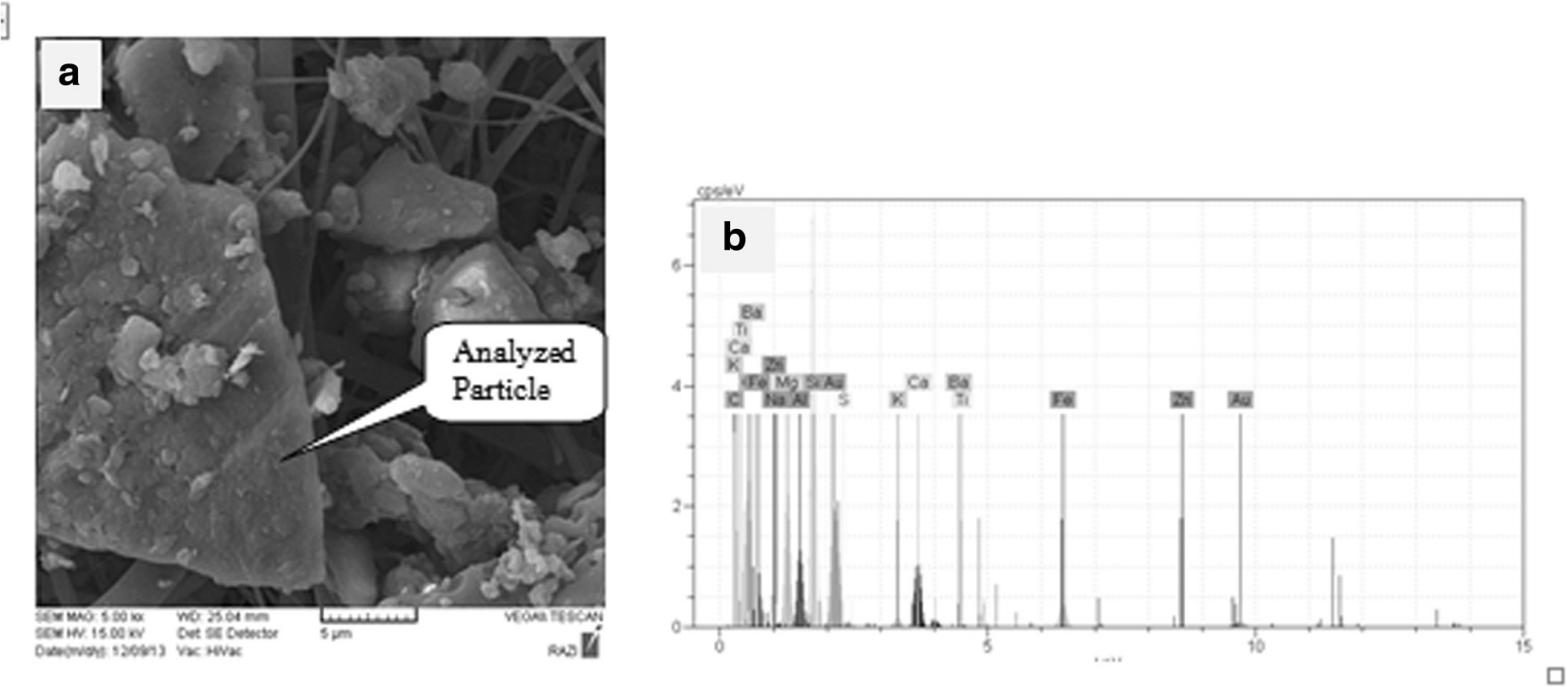
SEM photograph (**a**) and X-ray spectra (**b**) of TSP sample

**Fig. 6:**
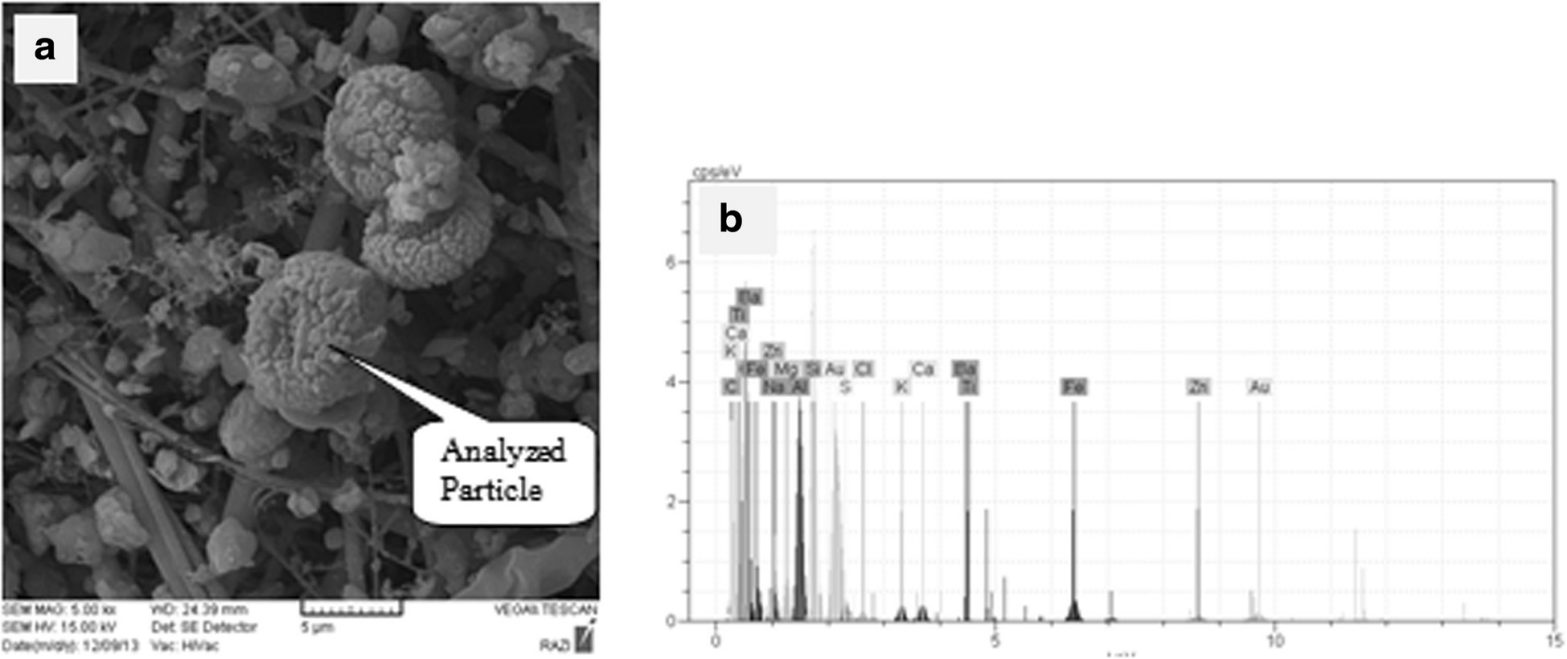
SEM photograph (**a**) and X-ray spectra (**b**) of PM_10_ sample

SEM-EDX photographs for TSP and PM_10_ samples are shown as an example in Figs. 5(a) and 6(a); and the SEM-EDX spectra of those are shown in Figs. 5(b) and 6 (b), respectively. Quantitative estimates of the particulate’s (TSP sample) elemental composition are given in Table 4. As shown in the table; Oxygen (30.7%) and Silicon (20.4%) are the major component of the analyzed particulate. However, other elements, such as Iron (8.7%), Magnesium (6.9%), Calcium (6.6%), Aluminum (4%), Sodium (1.2%), and Potassium (1%) are also of great importance. Also, elemental composition quantitative estimates of the PM_10_ sample are given in Table 5. It can be seen that Oxygen (29.1%) and Silicon (14.4%) are the major component of the analyzed particulate. However, other elements, such as Aluminum (8%), Zinc (7.1%), Iron (6.7%), Carbon (3.6%), Magnesium (1.7%), Chlorine (1.6%), and Sodium (1.3) are with great importance.

**Table 4:** Quantitative estimates of elemental compositions of TSP sample

Element	Series	unn. C [wt.-%]	norm. C [wt.-%]	Atom. C [at.-%]
Carbon	K series	1.01	1.05	2.4
Oxygen	K series	29.46	30.72	52.68
Sodium	K series	1.21	1.22	1.27
Magnesium	K series	6.61	6.89	7.78
Aluminium	K series	3.83	3.99	4.06
Silicon	K series	19.59	20.43	19.96
Sulfur	K series	0.33	0.34	0.29
Potassium	K series	0.95	0.99	0.7
Calcium	K series	6.35	6.63	4.54
Titanium	K series	0.08	0.09	0.05
Iron	K series	8.37	8.73	4.29
Zinc	K series	0.76	0.79	0.33
Barium	L series	0.01	0.01	0
Gold	M series	18.33	19.11	2.66
	Total:	95.90%

**Table 5:** Quantitative estimates of elemental compositions of PM_10_ sample

Element	Series	unn. C [wt.-%]	norm. C [wt.-%]	Atom. C [at.-%]
Carbon	K series	3.87	3.58	8.46
Oxygen	K series	31.51	29.15	51.66
Sodium	K series	1.42	1.31	1.62
Magnesium	K series	1.86	1.72	2.01
Aluminium	K series	8.93	8.26	8.68
Silicon	K series	15.58	14.41	14.55
Sulfur	K series	1.06	0.98	0.86
Chlorine	K series	0.61	0.57	0.45
Potassium	K series	1.33	1.23	0.89
Calcium	K series	1.27	1.17	0.83
Titanium	K series	0.13	0.12	0.07
Iron	K series	7.27	6.72	3.41
Zinc	K series	7.71	7.14	3.09
Barium	L series	0.01	0.01	0
	Total:	108.10%		

Comparison of the TSP and PM_10_ elemental composition with world average values revealed that amount of Silicon in the Urmia lake dusts is less rich (20.4% for TSP and 14.4% for PM_10_vs. the world average of 59.9%). However, the TSP resulted from Urmia lake dusts are richer in Fe (8.7% vs. the world average of 6.85%), Ca (6.6% vs. the world average of 3.94%), Mg (6.9% vs. the world average of 2.60%),and sodium (1.2% vs. the world average of 0.5–1%) than the Saharan, Harmattan, Chinese, and North American dusts [2].

It is important to note that the concentration of Chloride in the world average values is negligible; whereas our results indicated that chloride is one of the main ions in the Urmia lake dusts. This finding indicates that sea salt particles contribute large to the total mass of the particles collected. On the other hand, crustal dusts have also major role in the composition of TSP and PM_10_ in Urmia lake region.

### Ionic balance of PM

**Fig. 7:**
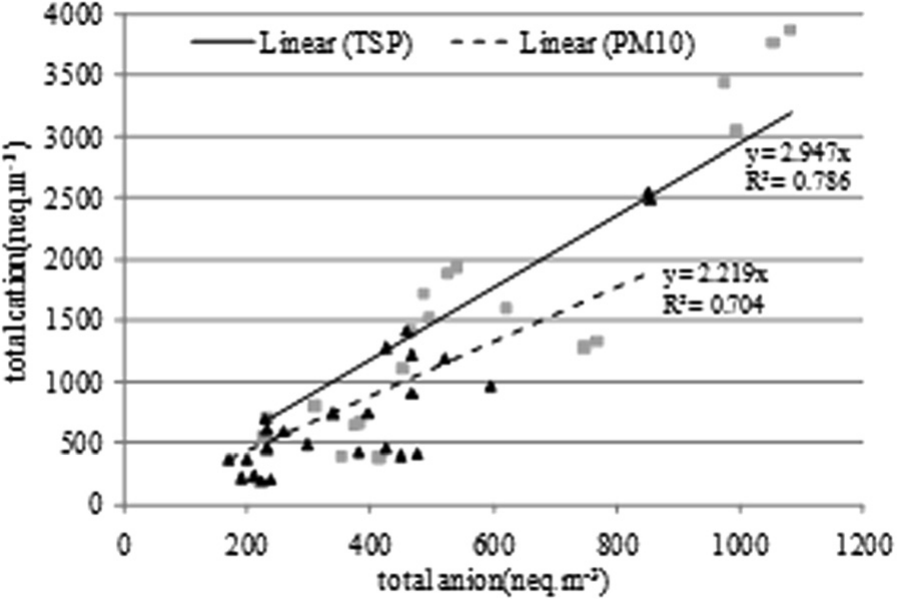
The ionic balance for TSP and PM_10_ fractions in Urmia lake region

Often the ionic balance is employed to determine the potentially ions missing, which are not measured during the extraction and the detection. The plots of total cations versus the total anions equivalents (*neq/m*^*3*^) are presented in Fig. 7. The slope of the regression line for PM indicated a value greater than unity (slope = 2.95, R^2^ = 0.79 for TSP and slope = 2.22, R^2^ = 0.7 for PM_10_), which might be due to high level of carbonate and bicarbonate, which were not analyzed. The main ions that could not be detected by our used method are HCO_3_^−^ and H^+^.

**Fig. 8:**
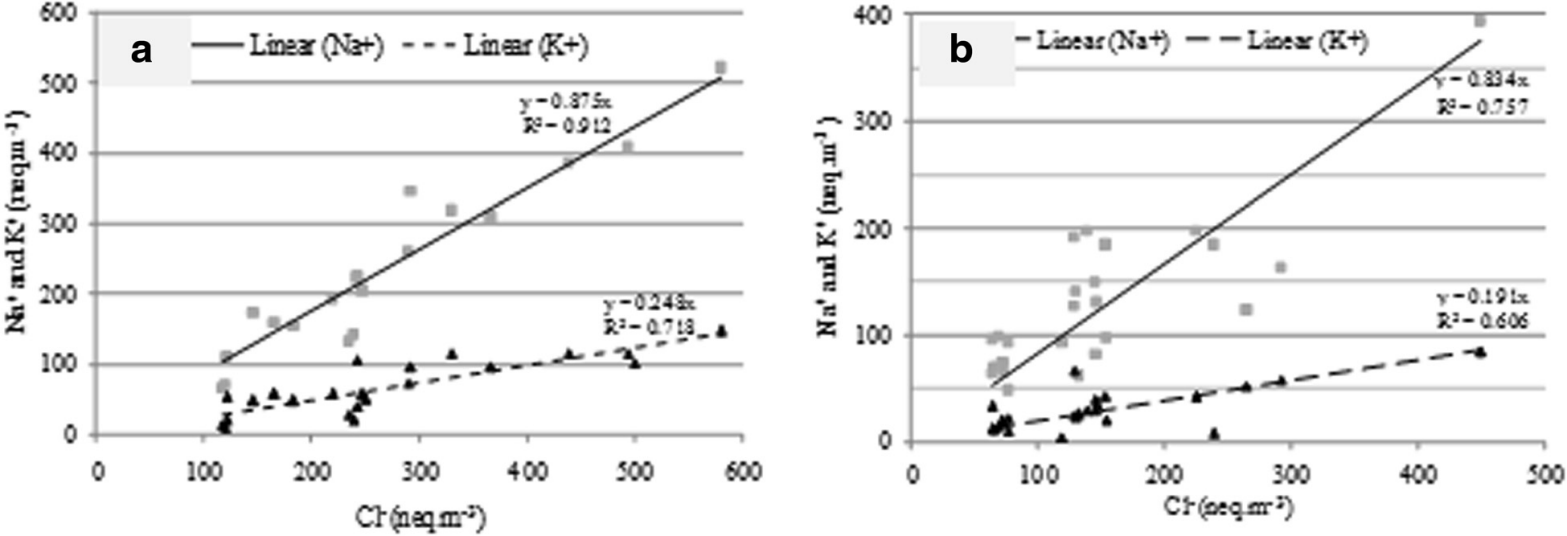
Relation between *Cl* ^−^with *Na* ^*+*^ and *K* ^+^ for (**a**) TSP and (**b**) PM_10_ fractions in Urmia lake region

On the other hand, as it was shown in Fig. 8, investigation of the relation between *Cl*^*−*^ with *Na*^+^ and *K*^*+*^ revealed strong correlation between *Cl*^*−*^ and *Na*^+^ (R^2^ = 0.91 and slope = 0.9 for TSP and R^2^ = 0.76 and slope = 0.8 for PM_10_) and *Cl*^*−*^ with *K*^+^. But, the slope of relation between *Cl*^*−*^ with *K*^+^ as smaller than *Na*^+^. These results allow us to conclude that the main chlorinated compound in PM was NaCl followed by KCl.

### Ionic correlation of PM

Ions correlation matrixes were calculated using bivariate correlations to quantify the relation between the elemental concentrations. As it is shown in Table 6, the number of significant correlations of the elements in PM_10_ is higher and also stronger than the TSP elements. Yatkin et al. [17] has stated that values of elemental correlations in PM_2.5_ were much higher than PM_10_. Therefore, it could be concluded that with decreasing the size of PM, the elemental correlation increases and it might show that the sources of smaller PM were limited compared to larger ones.

**Table 6:** The correlation matrixes of the elemental concentrations at urban site

	*Na* ^+^	*NH*_4_^+^	*K* ^+^	*Mg* ^2+^	*Ca* ^2+^	*F* ^−^	*Cl* ^−^	*NO*_2_^−^	*NO*_3_^−^	*SO*_4_^2 −^
	**TSP**									
*NH*_4_^+^	**0.593** ^**^									
*K* ^+^	**0.856** ^**^	**0.602** ^**^								
*Mg* ^2+^	0.482^*^	0.368	0.464^*^							
*Ca* ^2+^	0.344	0.014	0.363	**0.806** ^**^						
*F* ^−^	**0.657** ^**^	**0.658** ^**^	0.487^*^	0.396	0.096					
*Cl* ^−^	**0.956** ^**^	**0.692** ^**^	**0.848** ^**^	**0.546** ^**^	0.270	**0.550** ^**^				
*NO*_2_^−^	0.253	0.464^*^	0.178	**0.600** ^**^	0.407	0.397	0.256			
*NO*_3_^−^	**0.708** ^**^	0.347	**0.569** ^**^	0.504^*^	0.301	**0.925** ^**^	**0.576** ^**^	0.406^*^		
*SO*_4_^2 −^	**0.704** ^**^	0.457	0.547^**^	0.432^*^	0.266	**0.955** ^**^	**0.572** ^**^	0.444^*^	**0.958** ^**^	
*PO*_4_^2 −^	−0.053	0.194	−0.167	0.239	0.388	0.334	−0.090	0.485^*^	0.426^*^	0.503^*^
	**PM**_**10**_									
*NH*_4_^+^	**0.551** ^**^									
*K* ^+^	**0.749** ^**^	0.454^*^								
*Mg* ^2+^	**0.825** ^**^	**0.563** ^**^	**0.580** ^**^							
*Ca* ^2+^	**0.625** ^**^	0.257	0.441^*^	**0.875** ^**^						
*F* ^−^	**0.662** ^**^	0.187	**0.755** ^**^	0.547^**^	0.474^*^					
*Cl* ^−^	**0.882** ^**^	0.392	**0.795** ^**^	**0.814** ^**^	**0.753** ^**^	**0.834** ^**^				
*NO*_2_^−^	−0.087	0.345	−0.180	0.062	−0.120	−0.169	−0.293			
*NO*_3_^−^	**0.860** ^**^	0.316	**0.782** ^**^	**0.699** ^**^	**0.594** ^**^	**0.920** ^**^	**0.927** ^**^	−0.105		
*SO*_4_^2 −^	**0.868** ^**^	0.464^*^	**0.712** ^**^	**0.805** ^**^	**0.618** ^**^	**0.796** ^**^	**0.902** ^**^	0.031	**0.920** ^**^	
*PO*_4_^2 −^	−0.008	−0.386	0.211	0.019	0.190	0.549^**^	0.226	−0.199	0.306	0.030

^**^ *P* < 0.01 ^*^ *P* < 0.05

Bold numbers represented the significant correlation

High level of correlations was obtained between the terrestrial elements in TSP and PM_10_. For example, the correlation between *Ca*^2+^ and *Mg*^2+^ were 0.81 and 0.88 in TSP and PM_10_, respectively (*P* <0.01). These results suggest that the sources of the terrestrial elements are most probably soil and soil-related activities. Also, the high and strong correlation between *Cl*^*−*^, *Na*^+^, *Ca*^+^, *Mg*^2+^ and *K*^+^ indicates that a main part of PM was originated from marine salt. Correlations between *NH*_4_^+^ with *NO*_3_^−^ and SO_4_^2 −^ were weak; 0.35 and 0.46 in TSP and 0.31 and 0.46 for PM_10_, respectively (*p* > 0.05). It could be concluded that secondary ions have a few roles in the formation of TSP and PM_10_ in the Urmia lake region.

The *Cl*^*−*^/*Na*^+^ equivalent ratio for the whole study period ranged from 0.85, in warm seasons, to 1.76, in cold seasons, for TSP. This ratio for PM_10_ was 0.67, in warm seasons, to 2.15, in cold seasons. These results reveal that the loss of particulate *Cl*^*−*^ during warm seasons caused by the formation of gaseous *HCl* from *NaCl* and acidic gases [18]. The chloride loss may be illustrated by the reaction between HNO_3_/or H_2_SO_4_ and NaCl in sea-salt particles generally formulated by:

$$ HN{O}_3+ NaCl\ \left( sea\  salt\right)\to NaN{O}_3\left( sea\  salt\right)+HCl $$

The Cl^*−*^*/Na +* ratio in Urmia Lake region is broader and higher than the measured ratio in PM of urban and industrial zones of Tabriz (0.41–1.07 for TSP and 0.42–0.78 for PM_10_) [15].

### Principal components analysis of PM

Three air quality principal components (PCs) for TSP and two PCs for PM_10_ with eigenvalues exceeding 1.0 were found. Based on Table 7, it was indicated that these PCs have a significant influence on the PM quality in Urmia Lake. For the TSP, the first PC showed high loading of Na^+^*,* NH_4_^+^, K^+^, Cl^−^, F^−^, NO_3_^−^, and *SO*_4_^2 −^ with a total variance of 57.6 %. These ions are saline particulates and formed from Urmia lake bed. The second PC showed the high loading of Mg^2+^ and Ca^2+^ with a total variance of 18.41%. Theses ions are typically associated with crustal materials in windblown dust and the resuspended dust from around lands. Finally, the third PC showed high loading of ***NO***_2_^−^ and PO_4_^2 −^ with a total variance of 13.08 %. Application of chemical fertilizer in agricultural lands around sampling sites could be the origin of third PC.

**Table 7:** Varimax rotated PCA loadings for water-soluble ions of TSP and PM_10_ in Urmia Lake

Variables	**TSP**			**PM**_**10**_	
	PC1	PC2	PC3	PC1	PC2
Na+	**0.905**	0.31	−0.125	**0.926**	0.122
NH_4_^+^	**0.801**	−0.196	0.392	0.539	**0.707**
K^+^	**0.881**	0.34	−0.184	**0.824**	−0.121
Mg^2+^	0.36	**0.803**	0.335	**0.879**	0.217
Ca^2+^	0.056	**0.952**	0.197	**0.743**	−0.047
F^−^	**0.85**	−0.03	0.456	**0.825**	−0.424
Cl^−^	**0.913**	0.246	−0.094	**0.967**	−0.171
NO_2_^−^	0.195	0.302	**0.777**	−0.082	**0.63**
NO_3_^−^	**0.806**	0.191	0.473	**0.934**	−0.204
SO_4_^2 −^	**0.819**	0.092	0.537	**0.947**	0.113
PO_4_^2 −^	−0.064	0.172	**0.897**	0.167	**0.808**
Eigenvalue	6.341	2.025	1.439	6.585	1.881
% of Variance	57.648	18.41	13.085	59.861	17.097
% of Cumulative	57.648	76.06	89.142	59.861	76.958

Bold numbers represented the significant correlation

As mentioned above, two PCs were found for PM_10_. The first PC showed high loading of Na^+^, K^+^, Mg^2+^, Ca^2+^, Cl^−^, F^*−*^, NO_3_^−^, and SO_4_^2 −^ with a total variance of 59.9 %. These ions typically associated with saline particulates and crustal materials in windblown dust and resuspended dust from Urmia lake bed and sampling sites around lands. The second PC showed high loading of NH_4_^+^, ***NO***_2_^−^ and PO_4_^2 −^ with a total variance of 17.1%. High loading of these ions allows us to conclude that the main source of second PC was application of chemical fertilizer in agricultural lands around sampling sites.

## Conclusion

The implemented prevention and control measures are mainly the diversion or conservation of water to recover dried lake beds to reduce wind erosion. However, water-saving potential is limited due to continuing social and economical development in arid regions. On the other hand, evaporative losses from lake surfaces are high because of intense sunshine, large water areas, shallow water depths, and vigorous evaporation from the water surface.

As mentioned above, the water level of Urmia Lake has been decreased up to 6 m during last decade. Along with decreasing of water level, a salt desert is created with area more than 5000 km^2^. According to the dynamic characteristics of dust, grains with diameters smaller than 10 μm can be transported by wind for several thousands of kilometers under common wind power conditions, and those with diameters of 10–20 μm can be transported for hundreds or up to several thousands of kilometers with wind speeds > 15 m/s. Therefore, at Urmia Lake region and especially during the warm season, wind could carry on saline dust to adjacent areas as far as hundreds of kilometers. The transported PM could damage agricultural lands, pollute the ecosystem and cause variety of disease in some state of Iran, Azerbaijan and other neighboring countries.
